# Blockade of the TIGIT-CD155/CD112 axis enhances functionality of NK-92 but not cytokine-induced memory-like NK cells toward CD155-expressing acute myeloid leukemia

**DOI:** 10.1007/s00262-024-03766-7

**Published:** 2024-07-05

**Authors:** Katharina Seel, Ronja Larissa Schirrmann, Daniel Stowitschek, Tamar Ioseliani, Lea Roiter, Alina Knierim, Maya C. André

**Affiliations:** 1 Department of Pediatric Hematology and Oncology, University Children´s Hospital, Eberhard Karls University, Hoppe-Seyler-Str.1, 72076 Tuebingen, Germany; 2 Division of Respiratory and Critical Care Medicine, University Children`s Hospital Basel, University of Basel, Basel, Switzerland

**Keywords:** Acute myeloid leukemia, Natural killer cells, NK-92 cell line, Checkpoint receptor inhibition, TIGIT

## Abstract

TIGIT is an alternative checkpoint receptor (CR) whose inhibition promotes Graft-versus-Leukemia effects of NK cells. Given the significant immune-permissiveness of NK cells circulating in acute myeloid leukemia (AML) patients, we asked whether adoptive transfer of activated NK cells would benefit from additional TIGIT-blockade. Hence, we characterized cytokine-induced memory-like (CIML)-NK cells and NK cell lines for the expression of inhibitory CRs. In addition, we analyzed the transcription of CR ligands in AML patients (CCLE and Beat AML 2.0 cohort) in silico and evaluated the efficacy of CR blockade using in vitro cytotoxicity assays, CD69, CD107a and IFN-γ expression. Alternative but not classical CRs were abundantly expressed on healthy donor NK cells and even further upregulated on CIML-NK cells. In line with our finding that CD155, one important TIGIT-ligand, is reliably expressed on AMLs, we show improved killing of CD155^+^-AML blasts by NK-92 but interestingly not CIML-NK cells in the presence of TIGIT-blockade. Additionally, our in silico data (*n* = 671) show that poor prognosis AML patients rather displayed a CD86^low^ CD112/CD155^high^ phenotype, whereas patients with a better outcome rather exhibited a CD86^high^ CD112/CD155^low^ phenotype. Collectively, our data evidence that the complex CR ligand expression profile on AML blasts may be one explanation for the intrinsic NK cell exhaustion observed in AML patients which might be overcome with adoptive NK-92 transfer in combination with TIGIT-blockade.

**Supplementary Information:**

The online version contains supplementary material available at 10.1007/s00262-024-03766-7.

## Introduction

Acute myeloid leukemia (AML) is characterized by severe immune-permissiveness, defined by a high amount of exhausted T cells, natural killer (NK) cells and regulatory T cells, as well as low numbers of T helper cells and a clearly immunosuppressive cytokine milieu. In this context, autologous NK cells of patients with AML (in the following named AML-NK cells) exhibit a verifiable impairment in their functionality to target and eliminate tumor cells [1–9]. One reason for this lack of efficacy and the considerable functional exhaustion of NK cells is the occurrence of immune escape mechanisms imparted by the expression of checkpoint receptors (CR) ligands on the surface of tumor cells. We know that NK cells express classical CRs such as the programmed death-1 (PD-1) and the cytotoxic T-lymphocyte-associated protein-4 (CTLA-4) receptor, but also alternative CRs such as T-cell immunoreceptor with immunoglobulin and ITIM domain (TIGIT), V-Set immunoglobulin domain suppressor of T-cell activation (VISTA), T-cell immunoglobulin mucin-3 (TIM-3), lymphocyte activation gene-3 (LAG-3) and B7-H3 (CD276) [10]. However, to date, it is unclear to what extent the classical and alternative CRs control NK cell functionality toward hematopoietic malignancies. Likewise, it has remained an unresolved problem how AML patients who are likely to respond to checkpoint inhibition could be identified and how the appropriate checkpoint receptor–ligand axis should be selected. In line with the recent failure of classical CR inhibitors such as CTLA-4 or PD-1/PD-L1 inhibitors to show convincing activity in myeloid malignancies [11, 12], it is currently assumed that additional checkpoints and/or pathways must exist that critically suppress efficient anti-leukemia immunity of NK cells.

We, therefore, sought to obtain a clearer understanding of the molecular and context-dependent mechanisms by which alternative as opposed to classical CRs control NK cell functionality toward AML. As we believed a functional NK cell compartment to be critical for an effective anti-tumor response [13], we focused on characterizing the role of CRs expressed on primary NK cells of healthy donors (HD) and here particularly cytokine-induced memory-like NK cells, as these cells do not only exhibit exquisite Graft-versus-Leukemia effects but also display a complex heterogeneous receptor repertoire [14, 15] and have not been studied in the context of CR expression. In addition, we decided on testing various NK cell lines all of which have recently been tested for experimental adoptive cell transfer.

We decided to focus on testing the functional consequences of TIGIT inhibition as this immunoreceptor is one of the alternative CRs whose function as an inhibitory receptor on NK cells is well-documented [16–18]. Together with the co-inhibitory receptor CD96 and the co-stimulatory receptor CD226, TIGIT belongs to the poliovirus receptor (PVR)/Nectin family. All three PVR/Nectin family members bind to multiple inhibitory receptors, one of them being CD155, expressed on antigen-presenting cells but importantly also on malignant hematopoietic cells [19, 20]. Several groups have shown that TIGIT importantly contributes to immune tolerance [22] which might be successfully overcome by blocking of TIGIT on T cells [23] but also on NK cells [16–18].

Our data evidence that classical CRs such as PD-1 and CTLA-4 are virtually absent on various forms of NK cells, whereas certain members of the PVR/Nectin family are abundantly expressed. In line with our finding that CD155, the ligand for TIGIT, is reliably expressed on AML blasts, TIGIT-blockade significantly promoted the functionality of NK-92 cells, but surprisingly not of CIML-NK cells toward the CD155-expressing AML cell lines Molm-13 and HL-60. Additionally, our data indicate that the relationship between classical and alternative CR ligands on AML blasts might contribute to the immune evasion and prognosis. Collectively, our data provide evidence of optimized target cell recognition of CD155-expressing AML by NK-92 cells in the presence of TIGIT inhibitory antibodies. Hence, we not only identify patient attributes but also suggest a potential innovative form of NK cell-mediated immune therapy for relapsing AML patients.

## Materials and methods

### Generation of cytokine-induced memory-like NK cells

Following informed consent, primary NK cells were isolated from healthy volunteer donors via density gradient centrifugation using BioColl (1.077 g/ml, Bio&Sell, Nürnberg, Germany) and negative selection (EasySep Human NK Cell Enrichment Kit, STEMCELL Technologies, Cologne, Germany). To convert naïve NK cells to CIML-NK cells, primary NK cells were pre-activated for 16 h (on day -1) with 10 ng/ml IL-12 (PeproTech, Hamburg, Germany) and 50 ng/ml IL-18 (Medical & Biological Laboratories, Tokyo, Japan) as described before [14]. Pre-activated CIML-NK cells and unprimed control NK cells were both cultured in “RPMI 1640 complete medium” (RPMI 1640 plus 10% fetal calf serum, 100 U/ml penicillin, 100 μg/ml streptomycin and 2-mM L-glutamine) supplemented with 10% human serum, 100 IU/ml IL-2 (Novartis, Nürnberg, Germany) and 1 ng/ml IL-15 (CellGenix, Freiburg, Germany) from day -1 to ensure survival until day 7 (d7). After 16 h, both NK cell preparations were washed and replaced with complete medium which was refreshed on days 3 and 6 of culture. As cultivation conditions resulted in a slight activation and expansion of CD3^+^ T and CD3^+^CD56^+^ NKT cells, preparations with a CD3^+^ cell content of more than 5% were additionally depleted of residual CD3^+^ cells on day 6 using the EasySep Human CD3 Positive Selection Kit II (STEMCELL Technologies, Cologne, Germany). On d7, unprimed and CIML-NK cells of each donor were frozen for later usage. As such, phenotypical data represent the individual donors both with unprimed and the corresponding CIML-NK cells.

### Cell lines

All cell lines were originally obtained from ATCC (Wesel, Germany), or the Leibniz-Institute DSMZ (Braunschweig, Germany) and passaged more than 50 times. HL-60, Molm-13 and Nalm-16 were cultured in “RPMI 1640 complete medium.” The NKL cell line was cultivated in “RPMI 1640 complete medium” supplemented with 10 ng/ml IL-2, whereas the KHYG-1 and NK-YS cells lines were cultivated in “RPMI 1640 complete medium” supplemented with 20 ng/ml IL-2. For the NK-92 cell line, we used MEM-alpha GlutaMAX with 20% fetal calf serum, 100 U/ml IL-2, 100 U/ml penicillin, 100 μg/ml streptomycin, 2 mM L-glutamine, 0.02 mM folic acid, 0.2 mM myo-inositol and 0.05 mM 2-mercaptoethanol. All cell lines were regularly tested for mycoplasma.

### Immune phenotyping

To stain NK cells/cell lines, we used a cocktail consisting of CD56-BUV737 (NCAM16.2), CD3-APC-Cy7 (SK7), CD279 (PD-1)-PE-Cy7 (EH12.1), CD152 (CTLA-4)-PE-CF594 (BNI3), TIGIT-BB700 (741,182), CD226 (DNAM-1)-AF647 (DX11) and CD96 (TACTILE)-BV421 (6F9). To stain tumor cell lines, we used a cocktail consisting of CD274 (PD-L1)-BB515 (MIH1), CD273 (PD-L2)-APC-R700 (MIH18), CD80-BUV737 (L307.4), CD86-PE-Cy7 (2331 (FUN-1)), CD155 (PVR)-BV421 (SKII.4) and CD112-PE (TX31). All antibodies were purchased from BD Biosciences (Heidelberg, Germany) except for CD112-PE (TX31) which was obtained from BioLegend (San Diego, USA). For live–dead discrimination, we used ARD (Amine reactive dye) succinimidyl ester AF350 (A10168) from Thermo Fisher (Karlsruhe, Germany). The phenotyping was performed with a BD LSR II flow cytometer (BD Biosciences, Heidelberg, Germany).

### Differential expression analysis of CR mRNA transcripts

For comparison of RPKM normalized (log_2_) transformed checkpoint mRNA expression, we consulted data from the 2019 Cancer Cell Line Encyclopedia [24]. All available samples were included and stratified based on their primary diagnosis at specimen acquisition: acute myeloid leukemia (AML) (*n* = 35 samples) and acute B-cell lymphoblastic leukemia (B-ALL) (*n* = 15 samples). Subsequently, the expression of PD-L1, PD-L2, CD80, CD86, CD155 and CD112 was analyzed.

### Kaplan–Meier plots

For survival analysis, we consulted the Beat AML 2.0 cohort dataset [25] and stratified all samples with fully documented clinical information and existent transcriptome data (*n* = 671) according to their Z-score mRNA expression. To this aim, the highest quartile (highest Z-score expression) and lowest quartile (lowest Z-score expression) for the expression of PD-L1, PD-L2, CD80, CD86, CD155 and CD112 were analyzed (singular and combined). Kaplan–Meier plots were generated with cancer genomics data sets through the cBio Portal [26, 27], accessing expression-correlated survival data provided by the Tyner group [25].

### Dimensionality reduction visualization

The RPKM (Reads Per Kilobase Million) normalized bulk RNA data were visualized through a two-dimensional t-SNE plot. The t-SNE coordinates were calculated using the R package “Rtsne,” and the resulting plot was generated using the “plotly” package for visualization. For each Beat AML 2.0 cohort sample with available transcriptome data (Vizome platform, http://vizome.org, *n* = 707), classical and alternative checkpoint expression were analyzed demonstrating variance. The t-SNE plot was generated using a perplexity score of 30 and a θ-value of 0.5, resulting in two components. Each sample was assigned a color corresponding to its survival status.

### Determination of in vitro cytotoxicity

Target cells were labeled with CFSE on the day before the assay and cultured overnight in the medium specified above. For Nalm-16, we used 4 µM CFSE and for Molm-13 und HL-60 5 µM CFSE. As effectors, we used unprimed HD-NK cells (d7) and CIML-NK cells (d7) which were thawed and cultivated overnight in the respective medium. As additional effectors, we used NK-92 cells directly out of the culture. Monoclonal blocking antibodies (TIGIT mAb, 25 µg/ml, A15153G, BioLegend, Amsterdam, Netherlands; PD-1 mAb, 10 µg/ml, EH12.2H7, BioLegend, Amsterdam, Netherlands) were added immediately prior to the beginning of the co-incubation period. All experiments were performed with three technical replicates at the ratios indicated in the respective Fig. Legend. For live–dead discrimination, cells were stained with ARD (Amine reactive dye) succinimidyl ester Pacific Blue (Life Technologies, Carlsbad, CA, USA). Cells were analyzed either directly or fixed with 0.5% PFA and analyzed on the following day. The specific lysis was calculated by subtracting the spontaneously occurring cell death: (%CFSE^+^ARD^+^ dead targets – %CFSE^+^ ARD^+^ spontaneously dead targets)/(100 − %CFSE^+^ ARD^+^ spontaneously dead targets) × 100%.

### Extended functional response staining—determination of degranulation, cytokine secretion and activation

CIML-NK cells or NK-92 cells were co-cultured with the respective target cells (CFSE-labeled HL-60) in the presence or absence of TIGIT and/or PD-1 blocking mAbs at the above-mentioned ratios and antibody concentrations. We decided to use PMA (BD Biosciences, Heidelberg, Germany) at very low concentration of 1 ng/mL as co-stimulation to avoid IL-12, IL-15 and/or IL-18 which had already been used in the process of generating CIML-NK cells. One h prior to the beginning of the co-culture, CD107a-BV421 (H4A3) was added to the effector cells. Six h after the initiation of the co-culture, cells were washed and stained with the surface antibody CD69-PE-Cy7 (FN50). Subsequently, cells were permeabilized and co-stained with the respective intracellular antibody IFN-γ-PE (B27). All antibodies were from BioLegend. Cells were analyzed either directly or fixed with 0.5% PFA and analyzed on the following day.

### Statistics

Statistical evaluation was performed using GraphPad Prism version 9 (La Jolla, California, USA) and the Student`s *t*-test for comparison of the paired data (comparison of unprimed and CIML-NK cells), the Mann–Whitney *U*-test (comparison of mRNA expression data) and the Mantel–Cox test (Kaplan–Meier plots). Correlation analyses of the subset analyses data were done using the Spearman’s rank correlation test. Significant values were defined as *p* ≤ 0.05.

## Results

**Fig. 1:**
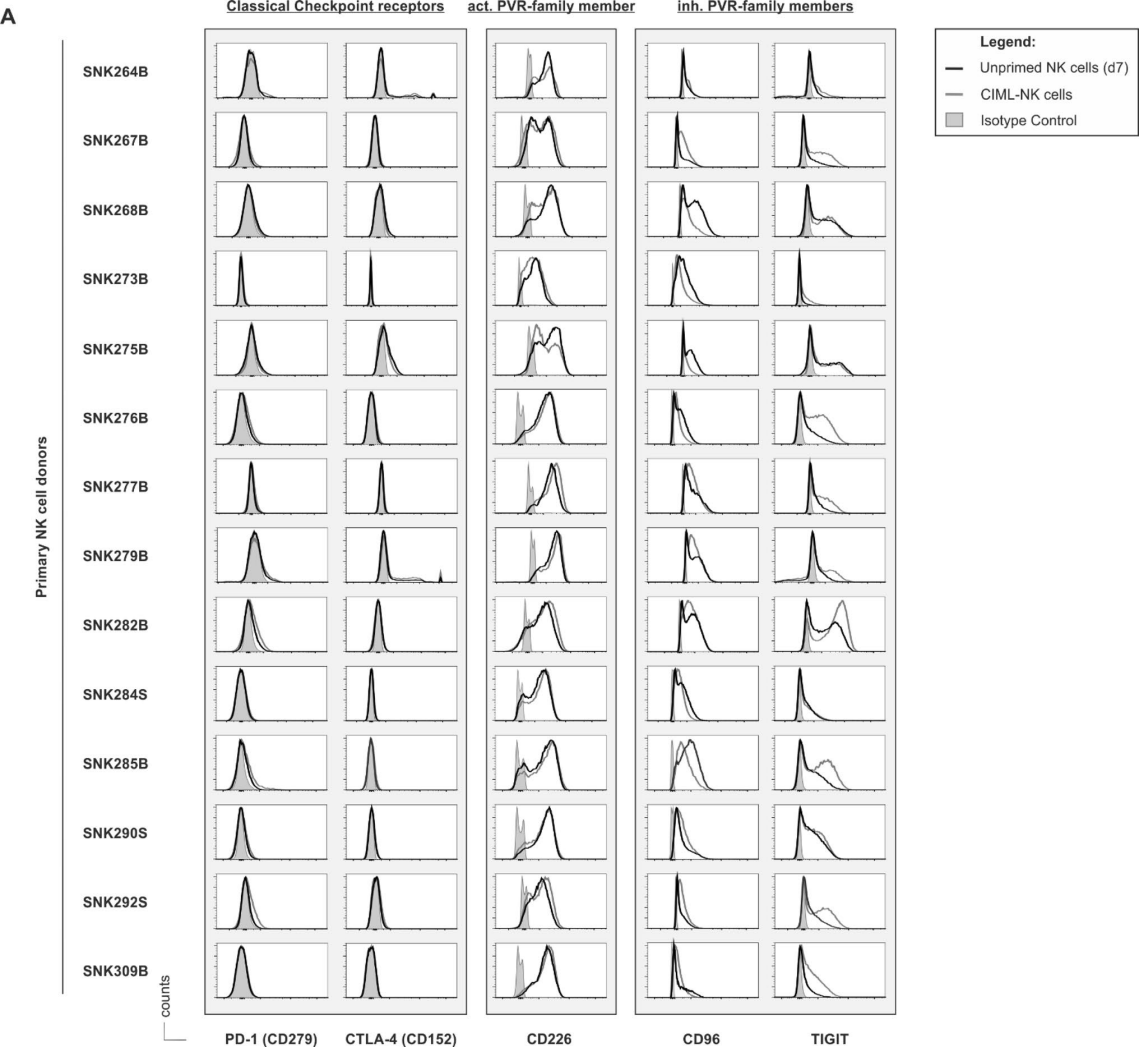

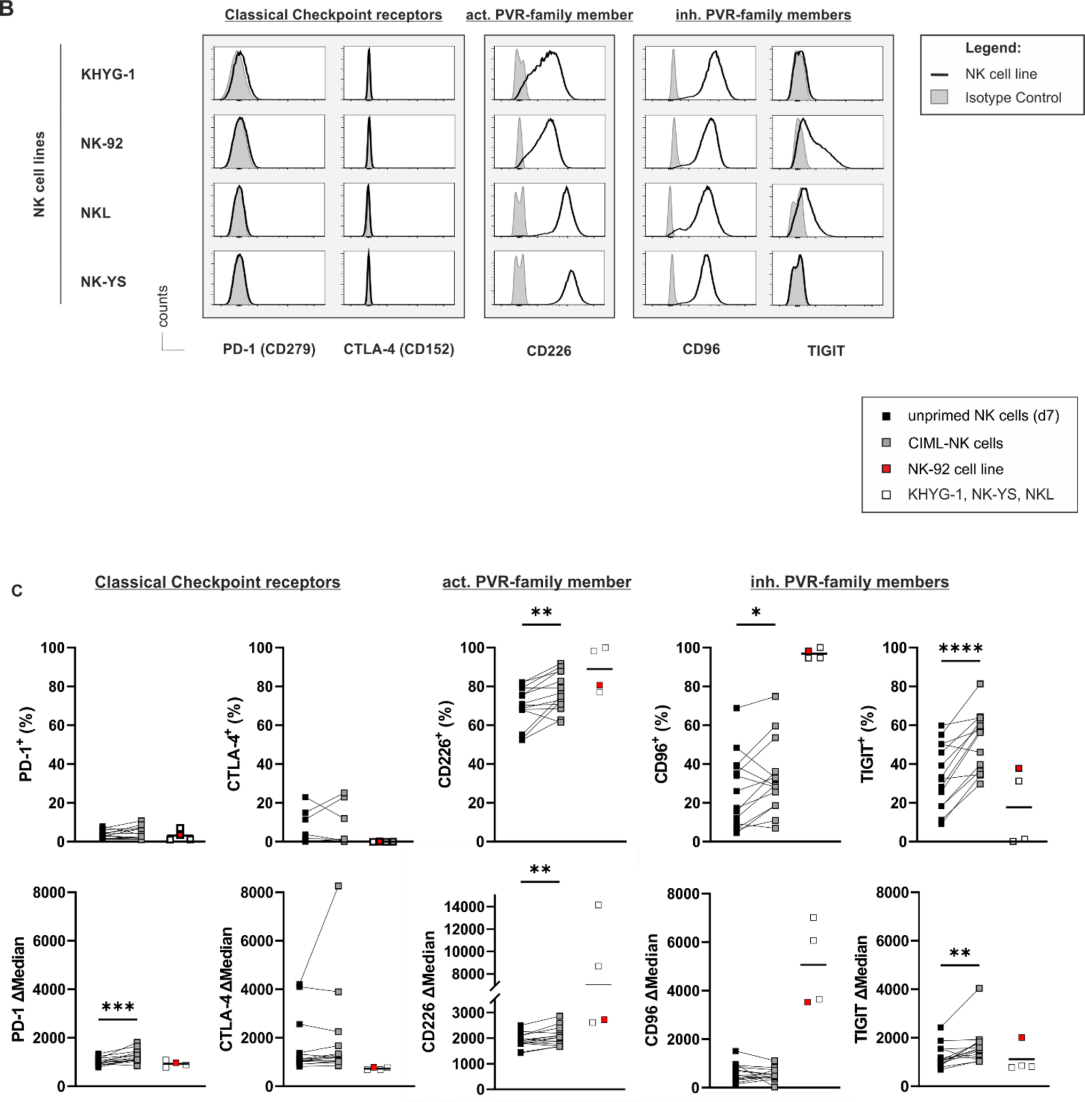
Unprimed and CIML-NK cells express PVR/Nectin family members but not classical immune CRs to a significant extent. Flow cytometric analyses of classical CRs (PD-1, CTLA-4) and activating (CD226) or inhibitory (CD96, TIGIT) alternative PVR/Nectin CRs on **A** unprimed NK cells (d7) and CIML-NK cells in comparison with **B** typical NK cell lines. **A** and **B** Overlay of the original histogram data displaying the respective antigen expression on unprimed NK cells or NK cell lines (black lines), respectively, or on CIML-NK cells (gray line), together with the corresponding isotype control (shaded in light gray). **C** NK cell subpopulation (%) and delta Median (ΔMedian) positive for one given CR. The ΔMedian was analyzed by subtracting the fluorescence intensity of the isotype staining from the specific staining of the respective antibody. Note that the process of memory cell conversion is accompanied by a significant upregulation of alternative but not classical CRs on CIML-NK cells. Each donor is represented with unprimed and CIML-NK cells. Statistical significance was calculated using the Student`s t-test for comparison of paired data and defined as * *p* ≤ 0.05, ** *p* ≤ 0.01, *** *p* ≤ 0.001 and **** *p* ≤ 0.0001

To obtain a clearer understanding of the mechanisms by which alternative as opposed to classical CRs control NK cell functionality toward AML, we performed immune CR profiling on various forms of NK cells (Fig. 1). To this aim, we selected unprimed HD-NK cells and their corresponding CIML-NK cells which have earlier been shown to promote exquisite anti-leukemic functionality against AML [14, 15] and acute B-cell precursor lymphoblastic leukemia (BCP-ALL) [28]. In addition, we included manufactural, off-the-shelve NK cell lines such as KHYG-1, NK-92, NKL and NK-YS which all have been discussed for experimental adoptive NK cell transfer [29].

In routinely used single receptor analyses, we found that classical CRs such as PD-1 and CTLA-4 were virtually absent on all NK cell preparations tested, whereas the PVR/Nectin family members TIGIT, CD96 and CD226 were expressed to a high extent on unprimed NK cells and CIML-NK cells (Fig. 1A, Suppl. Fig. 1A and Suppl. Fig. 2A and B). The same applied to NK cell lines, which lacked the expression of classical CRs but instead expressed alternative CRs (Fig. 1B, Suppl. Fig. 1A and Suppl. Fig. 2C and D). Interestingly, the process of memory NK cell conversion was accompanied by a clear upregulation of alternative (TIGIT, CD226 and CD96) but not of classical inhibitory CRs (PD-1 and CTLA-4). This was not only true for the size of the respective subset (indicated in %) but also for the intensity of receptor expression (indicated by the ΔMedian) (Fig. 1C).

**Fig. 2:**
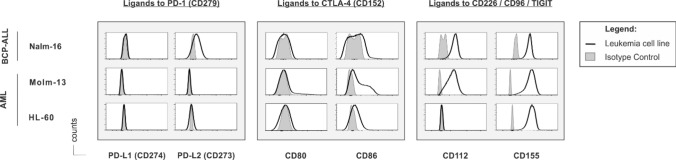
Acute lymphoblastic and myeloid leukemia cell lines express ligands to PVR/Nectin family receptors but not to classical CRs to a significant extent. Flow cytometric analyses showing the ligand expression to classical CRs (PD-L1 (CD274), PD-L2 (CD273), CD80 and CD86) but also to PVR/Nectin family members (CD112 and CD155) on the (pediatric) acute B-cell precursor lymphoblastic leukemia (BCP-ALL) cell line (Nalm-16) and two adult acute myeloid leukemia (AML) cell lines (Molm-13 and HL-60). Overlay of the original histogram data displaying the respective antigen expression (black lines) and the corresponding isotype control (shaded in light gray)

**Fig. 3:**
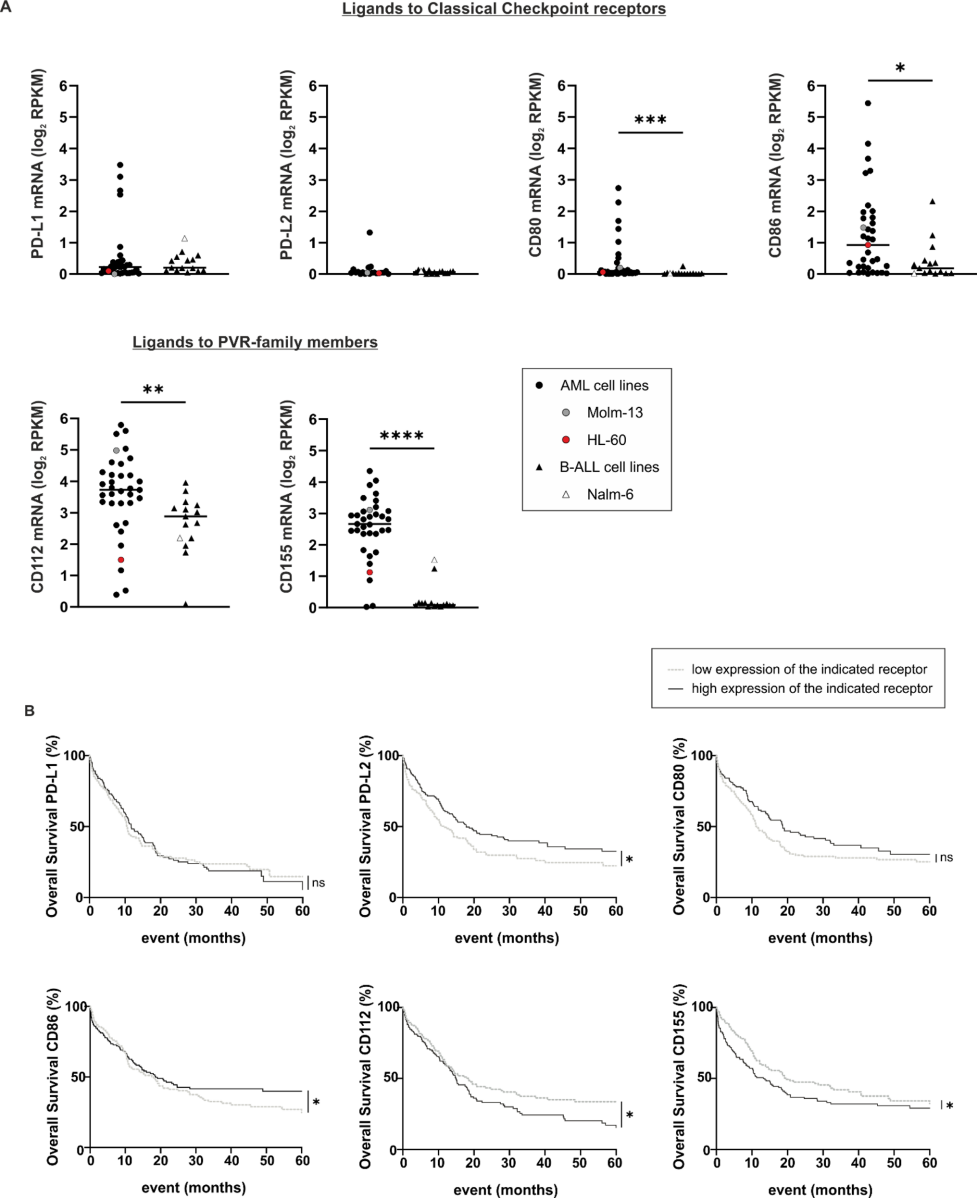

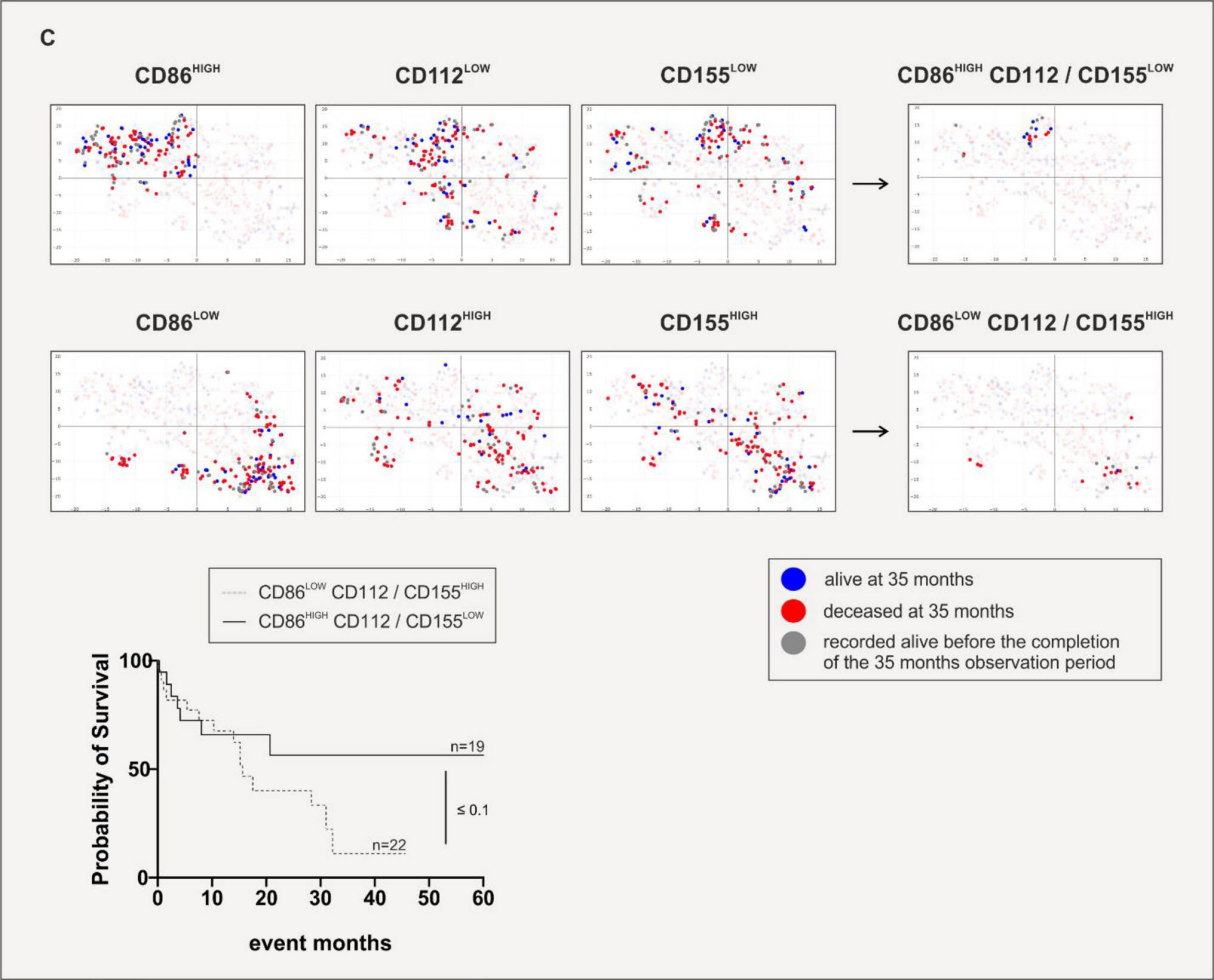
The complex alternative CR ligand profile on AML (but not B-ALL) blasts may have prognostic implications. For differential CR ligand mRNA expression analysis, the 2019 Cancer Cell Line Encyclopedia was frequented [24], and all available samples were included. Samples were stratified based on their primary diagnosis at specimen acquisition. **A** Differential mRNA expression of PD-L1, PD-L2, CD80, CD86, CD112 and CD155. Statistical significance was calculated using the Mann–Whitney U-test and defined as * *p* ≤ 0.05, ** *p* ≤ 0.01, *** *p* ≤ 0.001 and **** *p* ≤ 0.0001. Note that we show Nalm-6 as a related and similar substitute for Nalm-16 [30] in this Fig. as information on Nalm-16 was not available in the Encyclopedia. **B** Survival data of patients obtained from large-scale cancer genomics datasets through the cBio Portal [26, 27] as a function of high (black, solid line) or low (light gray, dashed line) mRNA expression of the respective CR ligand PD-L1, PD-L2, CD80, CD86, CD112 and CD155. Statistical significance was calculated using the Mantel–Cox test and was defined as * p ≤ 0.05. **C** Survival based on phenotype association. Two-dimensional t-SNEs plot of the AML patients in the Beat AML 2.0 cohort [25]. Each dot represents one specimen (*n* = 707). The normalized CR ligand mRNA transcripts (PD-L1, PD-L2, CD80, CD86, CD112 and CD155) demonstrating variance (with a perplexity score of 30 and a θ-value of 0.5) were subjected to t-SNE analysis. Patient samples are color-coded based on survival status at 35 months: “alive” (blue), “deceased” (red) and “recorded alive before completion of the 35 months observation period” (gray). Included is also a Kaplan–Meier plot showing that AML patients with a CD86^lo^ CD155/CD112^hi^ phenotype (light gray, dashed line) exhibit a poorer prognosis, whereas AML patients with a CD86^hi^ CD155/CD112^lo^ ligand profile (black, solid line) display an overall better outcome. Statistical significance was calculated using the Mantel–Cox test

As optimal checkpoint inhibition requires the expression of respective ligands on target cells, we subsequently characterized the expression of CR ligands on numerous leukemia cell lines and selected one B-ALL cell line (the pediatric B-cell precursor (BCP) acute lymphatic leukemia cell line Nalm-16) and two AML cell lines (Molm-13 and HL-60) as model cell lines for further analysis. Interestingly, all leukemia cell lines expressed ligands to PVR/Nectin family members (CD112 and CD155), except for HL-60 showing only the expression of CD155. Ligands to classical CRs such as PD-L1 (CD274) and PD-L2 (CD273), or CD80 and CD86 were absent or less expressed than ligands to alternative CRs on all these cell lines (Fig. 2 and Suppl. Fig. 1B). To ensure the clinical relevance of our findings, we analyzed the transcription of the respective ligands in AML and B-ALL cell lines in silico using the 2019 Cancer Cell Line Encyclopedia [24]. This analysis convincingly demonstrated that CR expression is inherently different between AML and B-ALL cell lines (Fig. 3A). Specifically, ligands to all CRs, particularly CD155, were detectable on most AML cell lines, whereas most (all but two) B-ALL cell lines (one of them being Nalm-6 which is related and similar to Nalm-16 [30]) expressed ligands at low levels and lacked any CD155 expression (Fig. 3A, Suppl. Table 1 and Table 2). Collectively, these data identify PVR/Nectin family members as potentially important players in NK cell-to-AML crosstalk and suggest the PVR/PVR-ligand axes as interesting targets for CR inhibition.

Assuming that CR ligand expression might be an indirect biomarker of prognosis, we next proceeded to analyze survival rates as a function of CR ligand expression for all AML patients included in the Beat AML 2.0 cohort [25] (Fig. 3B). This identified PD-L2 and CD86 (ligands to classical CRs) but also CD155 and CD112 (ligands to alternative CRs) as potential prognostic biomarkers for the survival of AML patients. In line with the distinct expression of CD155 on AML samples, we found that a high expression of CD155 correlated with a more unfavorable prognosis in AML patients. Specifically, median 5-year overall survival was 19.63 months (95% CI: 14.72–40.67) for patients with low CD155 expression and 13.22 months (95% CI: 9.3–19.20) for patients with high expression, resulting in a hazard ratio (HR) of 1.34 (95% CI: 1.0–1.8) with a log-rank p value of ≤ 0.04 (Fig. 3B). In line with the notion that the regulation of classical and alternative CR ligands follows different control mechanisms, the two-dimensional t-SNE plot cluster analysis but also the Kaplan–Meier survival curves of AML patients with comparable clinical characteristics (Suppl. Table 3, Suppl. Fig. 3) indicated that AML patients with a poorer prognosis rather display a CD86^low^ CD112/CD155^high^ phenotype, whereas AML patients with an overall better outcome rather exhibited a CD86^high^ CD112/CD155^low^ phenotype (*p* ≤ 0.1, Fig. 3C). While this analysis does not yet reach statistical significance, the Kaplan–Meier plots of surviving and diseasing patients are clearly different. As this largest-to-date dataset on primary AML samples is still growing, our descriptive data might, therefore, guide future researchers when re-analyzing survival rates of patients with varying CR phenotypes.

**Fig. 4:**
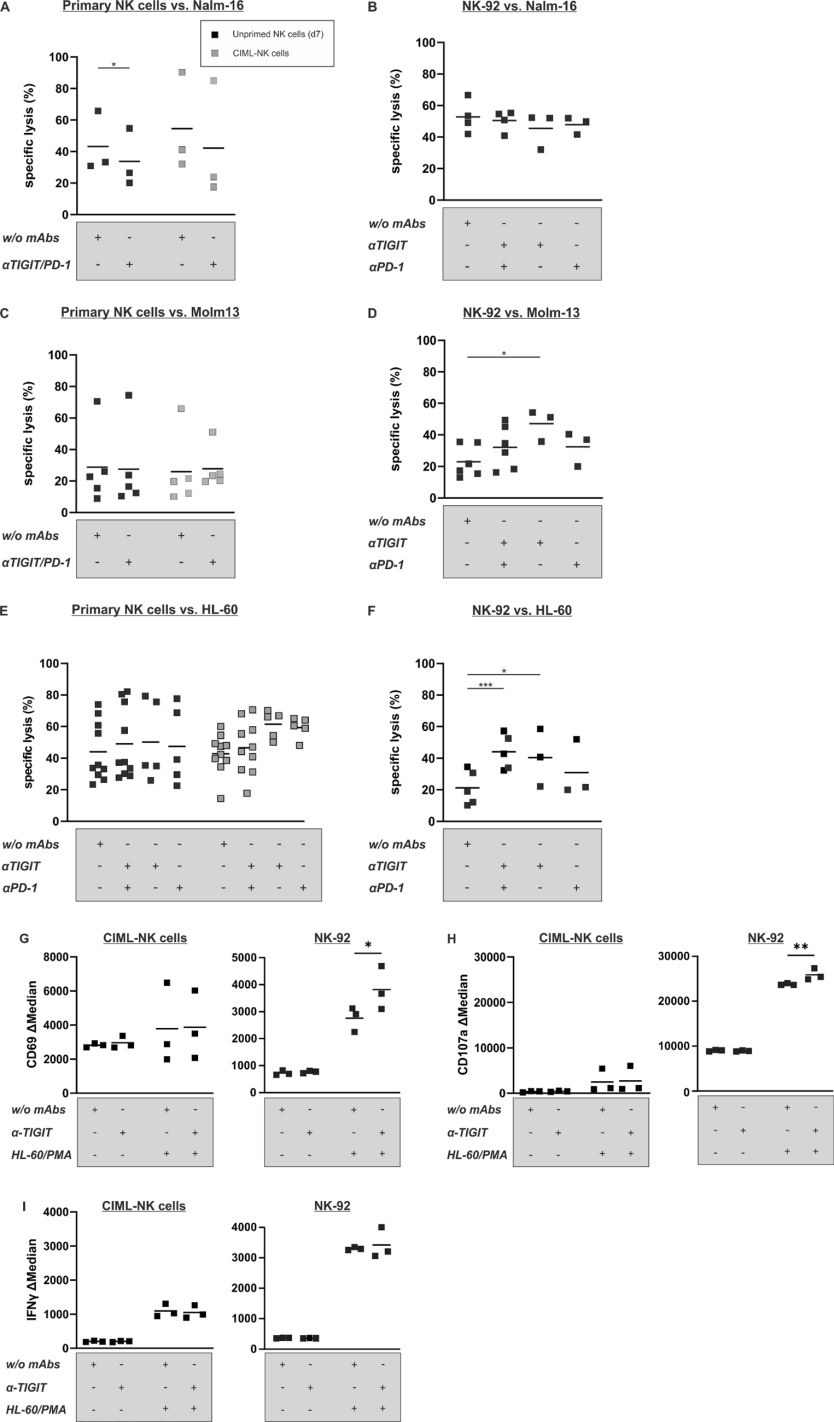
Anti-tumor functionality of the NK-92 cell line toward AML is enhanced by PVR/Nectin family member CR blockade. In vitro cytotoxicity was determined using unprimed NK cells (d7) and corresponding CIML-NK cells (d7) from healthy NK cell donors **A**, **C** and **E** or the NK-92 cell line **B**, **D** and **F** as effectors and Nalm-16, Molm-13 and HL-60 as targets. The following donors were used: testing against Nalm-16: SNK267B, SNK282B, SNK276B, testing against Molm-13: SNK285B, SNK267B, SNK292S, SNK309B, SNK268B and testing against HL-60: SNK275B, SNK268B, SNK309B, SNK264B, SNK279B, SNK313R, SNK314S, SNK294B, SNK295B, SNK278B, SNK304B. Please note that not all donors were tested in every single experimental condition as cell numbers were limited. The following effector-to-target (E:T) ratios were used: primary NK cells vs. Nalm-16 20:1, primary NK cells vs. Molm-13 20:1, primary NK cells vs. HL-60 5:1, NK-92 vs. Nalm-16 3:1, NK-92 vs. Molm-13 10:1 and NK-92 vs. HL-60 1:1. Experiments were performed in the absence or presence of PD-1 (EH12.2H7, 10 μg/ml)- and/or TIGIT (A15153G, 25 μg/ml)-blocking mAbs. Data represent *n* = 3–8 healthy NK cell donors and *n* = 3–6 independent experiments with the NK-92 cell line as effector. All experiments were performed in triplicates. (**G**–**I**) Extended functionality testing in co-culture experiments of CIML-NK cells of the donors SNK307B, SNK304B and SNK330L, NK-92 cells and HL-60. Functionality was determined in the presence or absence of TIGIT-blocking mAb (A15153G, 25 μg/ml) using the CIML-NK cells or the NK-92 cell line, respectively, as effectors and HL-60 as target cell line (ratio 1:1). **G** Level of activation as determined by CD69 expression. **H** Ability for degranulation as determined by intracellular CD107a expression. (**I**) Ability for cytokine secretion as determined by intracellular IFN-γ expression. Shown is the ΔMedian. Data represent *n* = 3 independent experiments. Significances in all experiments are indicated with * *p* < 0.05 and *** *p* < 0.001

We next proceeded to testing the functional consequences of blocking the TIGIT-CD155/CD112 axis (Fig. 4). As the TIGIT-binding monoclonal antibodies (mAbs) A15153A (BioLegend) and MBSA43 (Thermo Fisher) did not show reliable functional efficacy in our hands, we performed all subsequent experiments with the A15153G (BioLegend) mAb [31]. Additionally, we included the PD-1 blocking mAb EH12.2H7 (BioLegend) into our testing as several ex vivo studies recently suggested that blockade of TIGIT in the tumor microenvironment synergizes with the blockade of other CR inhibitors [32–34]. As effector cells, we used CIML-NK cells and the NK-92 cell line for our functional experiments as this cell line displayed the highest TIGIT expression of all NK cell lines tested, but more importantly as this cell line displays a proven high cytotoxicity against human leukemic cell lines and primary leukemia as well as superior cytotoxic activity compared to HD-NK cells in vitro and in mice [35]. Additionally, the NK-92 cell line has entered clinical trials for different malignancies including hematological malignancies [29].

In contrast with our expectations, we did not see any effect of the TIGIT-blocking mAb on the cytotoxic function of unprimed or CIML-NK cells (Fig. 4A, C and E). However, we did observe a significantly enhanced cytotoxicity of the NK-92 cell line (but not the KHYG-1, NKL and NK-YS cell line, data not shown) toward the AML cell lines Molm-13 and HL-60 but not the pediatric BCP-ALL cell line Nalm-16 in the presence of the TIGIT-blocking mAb (*p* ≤ 0.05) (Fig. 4B, D and F). This enhanced functionality of NK-92 cells (but not NKL cells, data not shown) correlated well with signs of increased activation (as determined by CD69 expression) and ability for degranulation (as determined by CD107a expression) (Fig. 4G and H), whereas the already maximal IFN-γ synthesis was not to be further increased (Fig. 4I). In line with the notion that our NK cell preparations and the selected hematopoietic malignant cell lines did not express PD-1 or PD-L1/2, respectively, we did not see a clear effect of additional PD-1-blockade in any of the various test combinations.

**Fig. 5:**
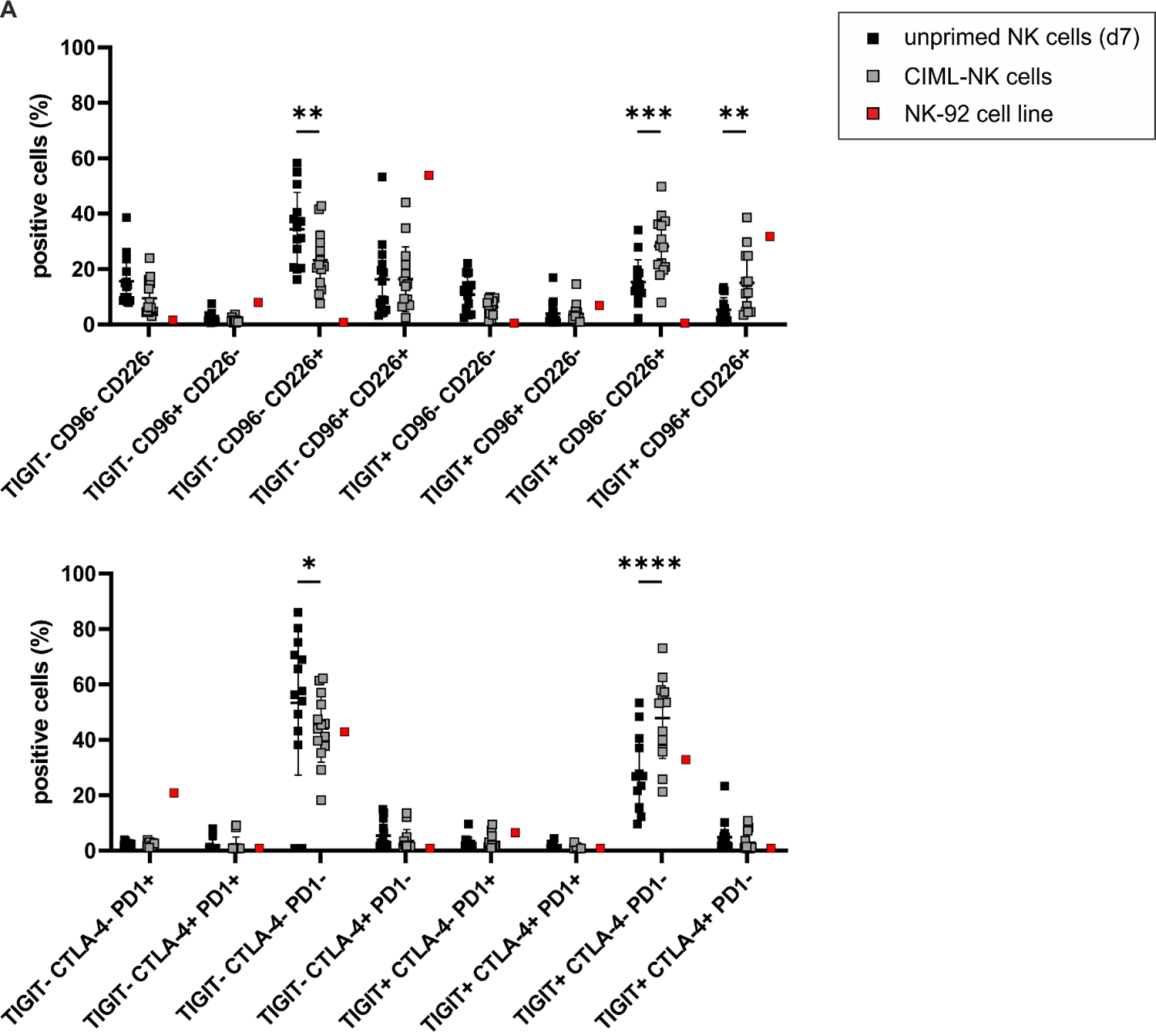
Subset analysis of alternative CR expression on unprimed and CIML-NK cells, and on NK-92 cells. Based on data of the flow cytometric analyses illustrated in Fig. 1C, the size of the individual subsets which are positive for a given combination of receptors is shown. Note that the process of memory cell conversion is accompanied by a significant upregulation of the double (TIGIT^+^ CD96^−^ CD226^+^) and triple (TIGIT^+^ CD96^+^ CD226^+^) positive NK cell subset at the expense of the single (TIGIT^−^ CD96^−^ CD226.^+^) positive subset. Significances as calculated with the Student`s t-test for comparison of paired data are indicated with * *p* < 0.05, ** *p* < 0.01 and *** *p* < 0.001

To potentially explain the finding that the TIGIT-blockade failed to improve the functionality of CIML-NK cells, we next performed a complex NK cell subset analysis knowing that NK cell subsets may vary considerably with respect to their functional efficacy depending on the individual subset composition of activating and inhibitory receptors. In line with findings on long-term activated and expanded NK cells [36], we showed that the size of the double (TIGIT^+^ CD96^−^ CD226^+^) and triple (TIGIT^+^ CD96^+^ CD226^+^) positive NK cell subsets was clearly increased during memory cell conversion at the expense of the single (TIGIT^−^ CD96^−^ CD226^+^) positive subset (Fig. 5A). Given that the characterization of CIML-NK cells has to date largely been confined to describing activation or maturation, it is interesting to note that IL-12/15/18 cytokine exposure obviously also induces upregulation of inhibitory checkpoint receptors, such as TIGIT and CD96. In contrast, NK-92 cells displayed a monomorphic CR expression profile comprising only of two inhibitory alternative CRs subsets, namely, the TIGIT^−^ CD96^+^ CD226^+^ and the TIGIT^+^ CD96^+^ CD226^+^ subset (Fig. 5).

In line with the fact that CIML-NK cells express a highly activated CD94^+^NKG2C^+^CD69^+^CD57^−^KIR^−^ receptor expression profile [14], we conclude that despite the upregulation of TIGIT on CIML-NK cells, these are obviously little “exhausted.” As such, the functionality of CIML-NK cells is probably not relevantly controlled by the expression of TIGIT itself. In contrast, long-term cultured and monomorphic NK-92 cells obviously bear—despite an intrinsically high level of activation—inhibitory receptors that participate in controlling functionality. We suggest that this may result to some extent in an “exhaustion phenotype,” i.e., expression of inhibitory checkpoint receptors that will respond toward TIGIT-blockade when tested against CD155^+^-AML cell lines. Collectively, our data suggest that the TIGIT-CD155/CD112-signaling pathway may indeed have a role in the evasion of AML from the innate immune system. Here, it appears that the adoptive transfer of NK-92 cells combined with TIGIT-blockade might prove to be beneficial for CD155-expressing, relapsing AML patients.

## Discussion

Using immune CR profiling on unprimed NK cells, CIML-NK cells and various NK cell lines, we here demonstrate that classical CRs such as PD-1 and CTLA-4 are virtually absent on all these preparations, whereas PVR/Nectin family members such as TIGIT, CD96 and CD226 are expressed to a significant extent (Fig. 1). This is in line with earlier findings describing that primary human NK cells lack the expression of classical but express alternative CRs [37]. More importantly, it is interesting to note that the conversion of cytokine-induced memory-like NK cells is obviously associated with the upregulation of alternative inhibitory checkpoint receptors although CIML-NK cells are per se highly activated [14, 15]. The observation that any form of activation also induces the upregulation of counter-balancing inhibitory receptors has also been reported for short-term [36] and long-term activated and expanded NK cells [36, 38].

Somewhat unexpectedly, we observed that despite this upregulation of inhibitory CRs, the blockade of TIGIT did not result in a significantly higher cytotoxicity of CIML-NK cells toward leukemia (Fig. 4). In contrast with these findings in CIML-NK cells but in line with the previous findings reported by the Fiedler group [31], we observed a clear effect of TIGIT-blockade when testing the cytotoxicity of the NK-92 cells toward the AML cell lines Molm-13 and HL-60. Our in-depth analysis of the alternative CR receptor profile revealed that TIGIT is primarily upregulated on CD226^high^ CIML-NK cell subsets (Fig. 5). It, therefore, appears that next to the multitude of upregulated activating receptors on CIML-NK cells such as CD94, CD69 and NKp46 but not killer immunoglublin-like receptors (KIRs) [14], the equilibrium of activating and inhibitory alternative CRs is also altered during the process of memory cell conversion. Given the considerable heterogeneity of TIGIT expression on CIML-NK cells and the limited number of NK cell donors tested in our functional assays, we at this point cannot fully exclude the possibility that CIML-NK cells of some individuals might indeed respond to TIGIT inhibition. However, accounting for this documented heterogeneity would make the generation of a GMP-compliant product for experimental adoptive immune transfer impossible. We, therefore, think that the possibility of adoptive CIML-NK cell transfer plus TIGIT-blockade is unlikely to be successful in the clinic.

Interestingly, TIGIT-blockade did not affect NK-92-mediated lysis of the pediatric BCP-ALL cell line Nalm-16. While the reasons for this remain unclear, it is interesting to note that the Mezger group likewise described that a CRISPR-Cas9-guided knockout of TIGIT promotes functionality of CAR-bearing NK-92 cell preparations only toward AML (i.e., U937) but not toward B-cell precursor (BCP)-ALL (i.e., Nalm-6) [39]. In line with the notion that fundamental phenotypical differences exist between adult and pediatric B-cell precursor lymphatic leukemia with respect to cell adhesion molecules and MHC class I ligands [40–42], we currently assume that the functional significance of the TIGIT-CD112/CD155 axis might also significantly differ between AML and pediatric BCP-ALL.

**Fig. 6:**
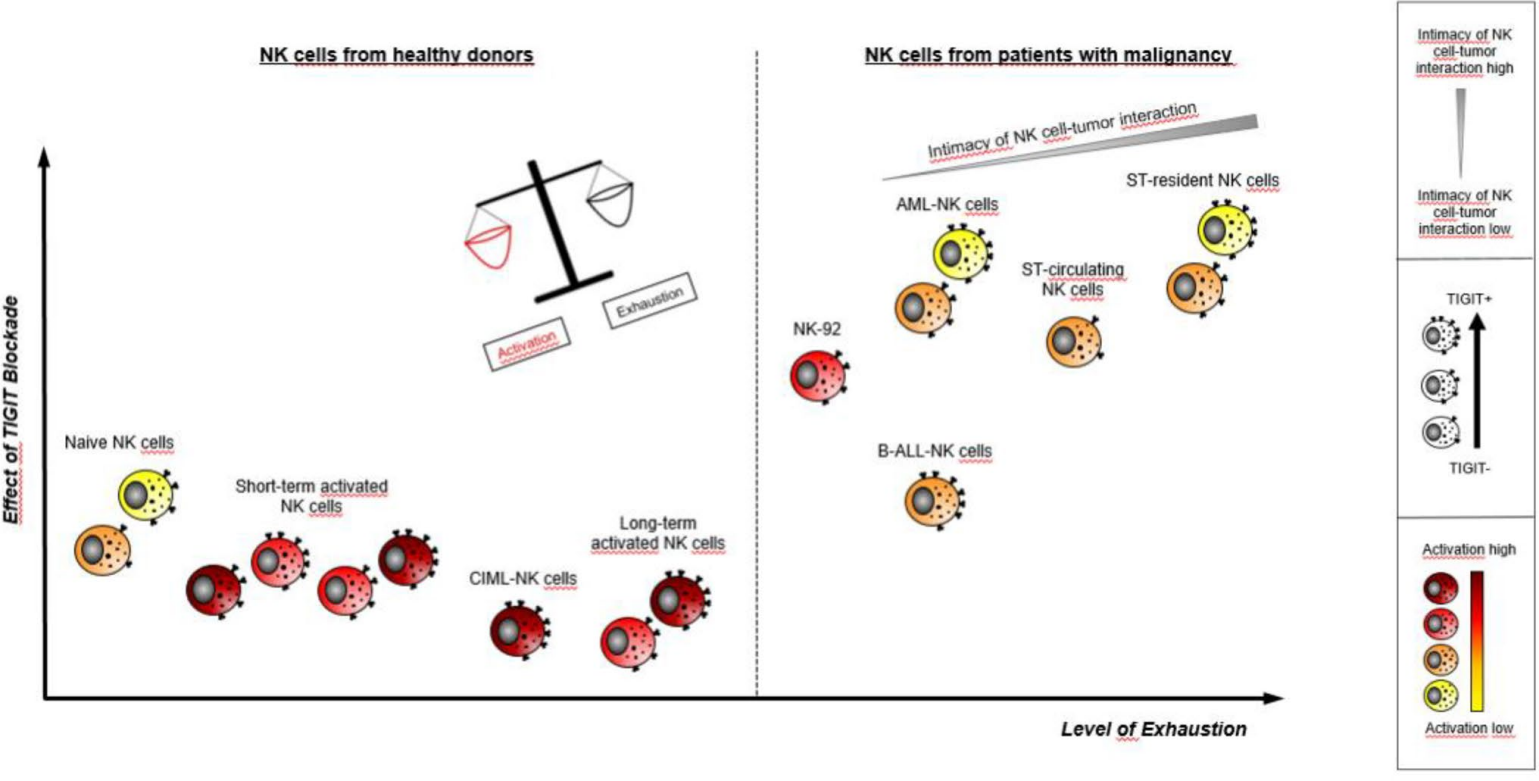
Hypothesis. The effect of a therapeutic TIGIT-blockade will depend on the balance between NK cell activation and exhaustion. This balance will be significantly different in healthy donors or tumor-bearing patients. The proximity of NK cells and tumor cells in the microenvironment of a solid tumor probably induces the highest level of functional exhaustion and as such the greatest likelihood to respond to therapeutic TIGIT-blockade. The group of healthy donor NK cells consists of unprimed NK cells, IL-2 or IL-12/15/18 short-term activated NK cells, CIML-NK cells and feeder-induced long-term activated NK cells. The group of NK cells from tumor-bearing patients comprises NK-92 cells, AML-NK cells, B-ALL-NK cells and solid tumor-resident or solid tumor-circulating NK cells. Note that the putative number of TIGIT receptors varies according to the assumed level of exhaustion. Activation levels are marked in varying shades of yellow to red. Indicated is also the increasing level of cell–cell contact between NK cells and different forms of tumor disease. ST: solid tumor

Lately, it has been appreciated that pathways controlling the activation and exhaustion are not necessarily mutually exclusive [43]. Hence, NK cells may simultaneously express several major activating receptors such as CD226, natural killer group 2 member D (NKG2D), NKp46 and NKp30 but may also express inhibitory alternative CRs such as TIGIT. While the activating CD25^++/+++^ CD69^++^ NKp30^++^ NKp44^+^ NKp46^++^ NKG2D^+++^ NKG2C^+^ phenotype of CIML-NK cells has been described by us [28, 44] and others [15], comparatively little is known about the inhibitory phenotype, and particularly about the relative contribution of both arms to the functionality of CIML-NK cells. In long-term cultivated, highly activated (PM-21) NK cells, it has been described that CD226-CD155 binding and/or triggering through the activating natural killer group 2 member D (NKG2D) receptor may overpower TIGIT inhibitory signaling [36]. In addition, it has been shown that the cytokine IL-15 modulates the balance between activation and exhaustion by simultaneously promoting cytotoxic function and inducing the expression of inhibitory TIGIT on NK cells in patients with sarcomas [45]. At this point, we, therefore, hypothesize that the differences in the functional responsiveness toward TIGIT-blockade seen in CIML-NK cells and NK-92 cells reflect an altered balance between activating and inhibitory receptors resulting in overall differing degrees of functional exhaustion. Following this line of thought and acknowledging the fact that differences in the response toward CR inhibition also exist between patients with solid tumors and AML, it is tempting to speculate that the level of exhaustion must also be disparate for NK cells that reside localized in solid tumors, that circulate in the blood of patients with solid tumors or that are widely disseminated in patients with AML (Fig. 6). In this regard, gradual differences in the intimacy of the contact between tumor and NK cells probably account for varying levels of immune cell exhaustion, and presumably also for the varying clinical response to therapeutic TIGIT-blockade.

The importance of alternative CR ligand expression and the assumed NK cell exhaustion phenotype in AML patients is underlined by our in silico findings (Fig. 3). Strikingly, we found that more samples expressed ligands to PVR/Nectin family members (CD112 and CD155) than ligands to classical CRs such as PD-L1 (CD274) and PD-L2 (CD273), or CD80 and CD86. Moreover, a high expression of CD155 correlated with unfavorable prognosis in the AML patients included in the Beat AML 2.0 cohort. Interestingly, our analysis also demonstrated that AML patients with a CD86^low^ CD112/CD155^high^ phenotype rather displayed a poor prognosis, whereas AML patients with a CD86^high^ CD112/CD155^low^ AML phenotype exhibited a more favorable outcome. Despite the limited size of the patient cohort (*n* = 671) and the incomplete information that existed for some patients regarding their survival status, this potentially helpful phenotypic AML profile might guide future research and should, therefore, be validated in a prospective way in AML patients with varying cancer type or morphology.

Collectively, our study provides first in vitro evidence that the unique cytolytic properties of NK-92 cells could potentially be further improved by co-administration of TIGIT-blocking antibodies. While TIGIT-blockade might, therefore, have a role in adoptive NK cell transfer protocols using the NK-92 but not other NK cell lines, it is probably less relevant or ineffective in protocols using CIML-NK cells. Without doubt, AML is a very heterogeneous disease, and our data can only provide a first attempt to better characterize the immune-permissive landscape of AML. However, our observation that TIGIT inhibition may promote the anti-leukemic activity of NK-92 but not of CIML-NK cells raises the question of whether future composite biomarker analysis should not also include an in-depth functional assessment of the corresponding host immune cells, i.e., T and NK cells to enable a more comprehensive understanding of the intricate immune regulatory network of the PVR/Nectin family members, other immune CRs and their respective ligands on AML blasts.

## Supplementary Information

Below is the link to the electronic supplementary material.

Supplementary file1 (DOCX 1370 KB)

## Abbreviations

AML: Acute myeloid leukemia
B-ALL: Acute B-cell lymphoid leukemia
CIML-NK cells: Cytokine-induced memory-like NK cells
CR: Checkpoint receptor
CTLA-4: Cytotoxic T-lymphocyte-associated protein-4
GvL: Graft-versus-Leukemia
HD: Healthy donor
NK: Natural killer cells
NKG2D: Natural killer group 2 member D
PD-(L)1: Programmed death-(ligand)1
PVR: Poliovirus receptor
TIGIT: T-cell immunoreceptor with Ig and ITIM domains
