# Supplementary material for: Blockade of the TIGIT-CD155/CD112 axis enhances functionality of NK-92 but not cytokine-induced memory-like NK cells toward CD155-expressing acute myeloid leukemia
Source: Cancer Immunol Immunother. 2024 Jul 5;73(9):180. doi: 10.1007/s00262-024-03766-7 (PMC11226419; doi:10.1007/s00262-024-03766-7)
Supplement: Supplementary file 1 — Supplementary file1 (DOCX 1370 KB) [file 262_2024_3766_MOESM1_ESM.docx]

**Supplemental Data**

Suppl. Table 1. Selected AML cell lines used in the differential checkpoint mRNA expression analysis. Data were obtained from the 2019 Cancer Cell Line Encyclopedia [1]. N.A.: information missing.

Suppl. Table 2. Selected B-ALL cell lines used in the differential checkpoint mRNA expression analysis. Data were obtained from the 2019 Cancer Cell Line Encyclopedia [1]. N.A.: information missing.

Suppl. Table 3. Clinical data of AML patients included in Fig. 3c. Data were obtained from the Beat AML 2.0 cohort dataset [1] and accessed via the cBioPortal [2]**.** p-values indicate significances between AML patients with a CD86^low^ CD112/155^high^ vs. CD86^high^ CD112/CD155^low^ phenotype as determined with Chi-squared- or Wilcoxon Test. Note, that differences only exist for the cancer type and morphology but not for clinical parameters that might serve as potential confounders.

Suppl. Table 3. Continued.

**Supplemental Figures**

**
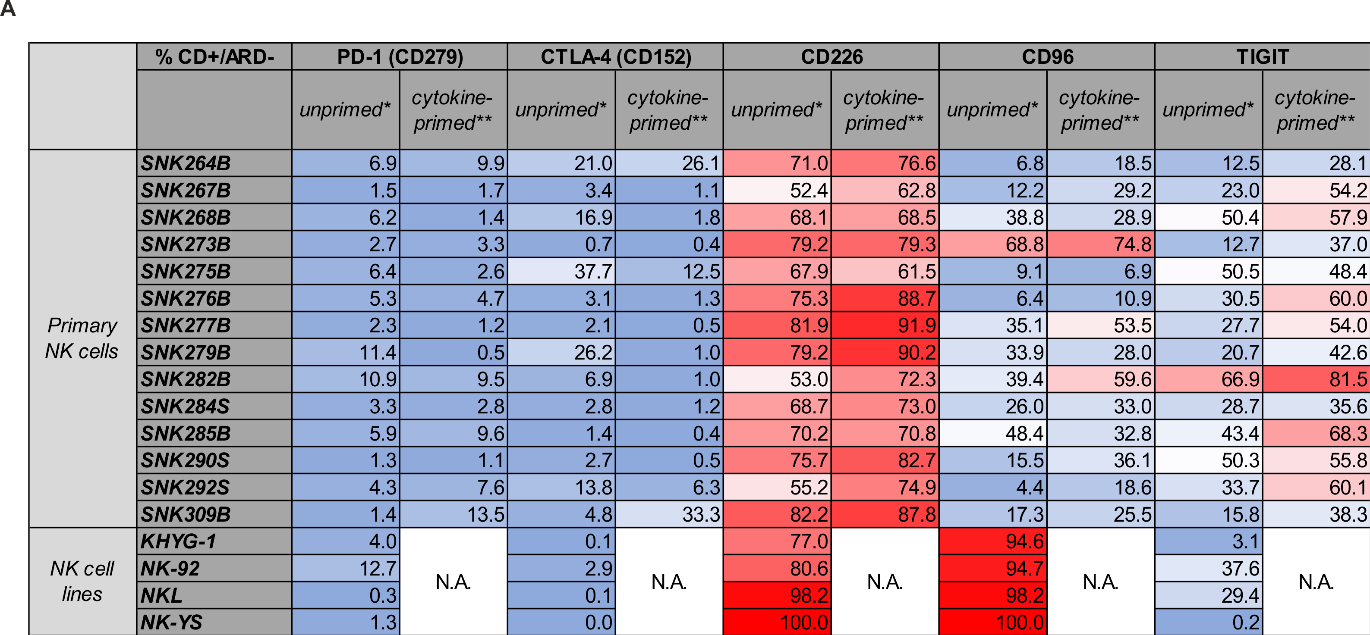
**

* corresponds to either NK cell lines or unprimed NK cells (d7) as indicated in the first column

** corresponds to cytokine-induced memory-like (CIML)-NK cells

**
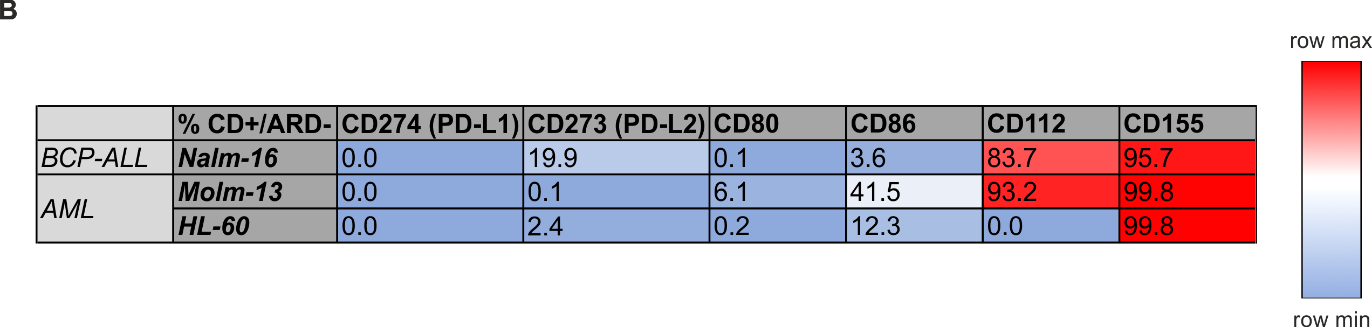
**

Suppl. Fig 1. NK cell preparations and leukemia cell lines of various origins express rather alternative than classical CRs / CR ligands, respectively. **A** Heat mapping with continuous blue-white-red shading. Colors vary continuously in shades of blue (below control median) or red (above control median) with an intensity proportional to the difference from the median (0.5 centile). **B** Heat mapping in acute lymphoblastic or myeloid leukemia cell lines using continuous blue-white-red shading. Colors vary continuously in shades of blue (below control median) or red (above control median) with an intensity proportional to the difference from the median (0.5 centile).


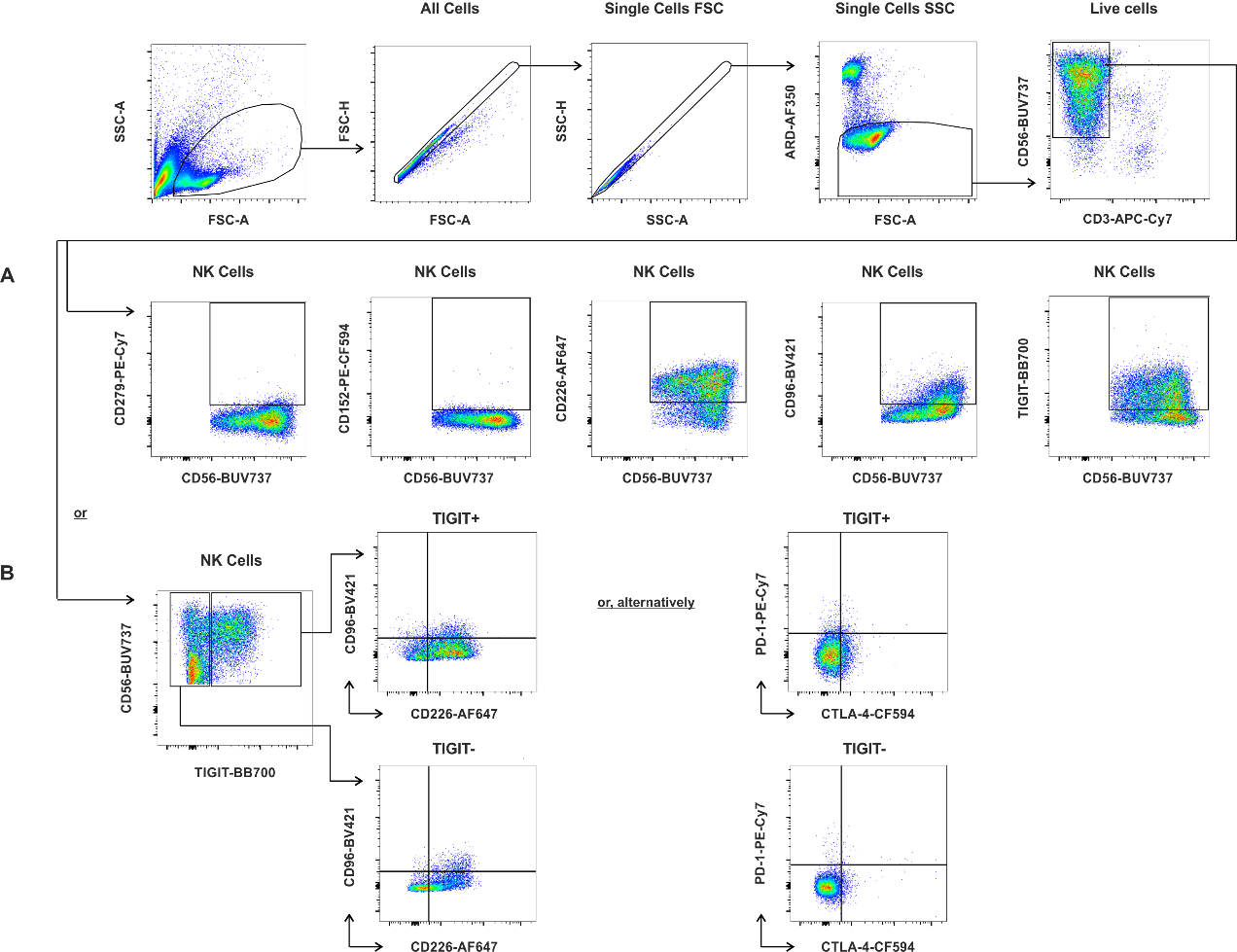


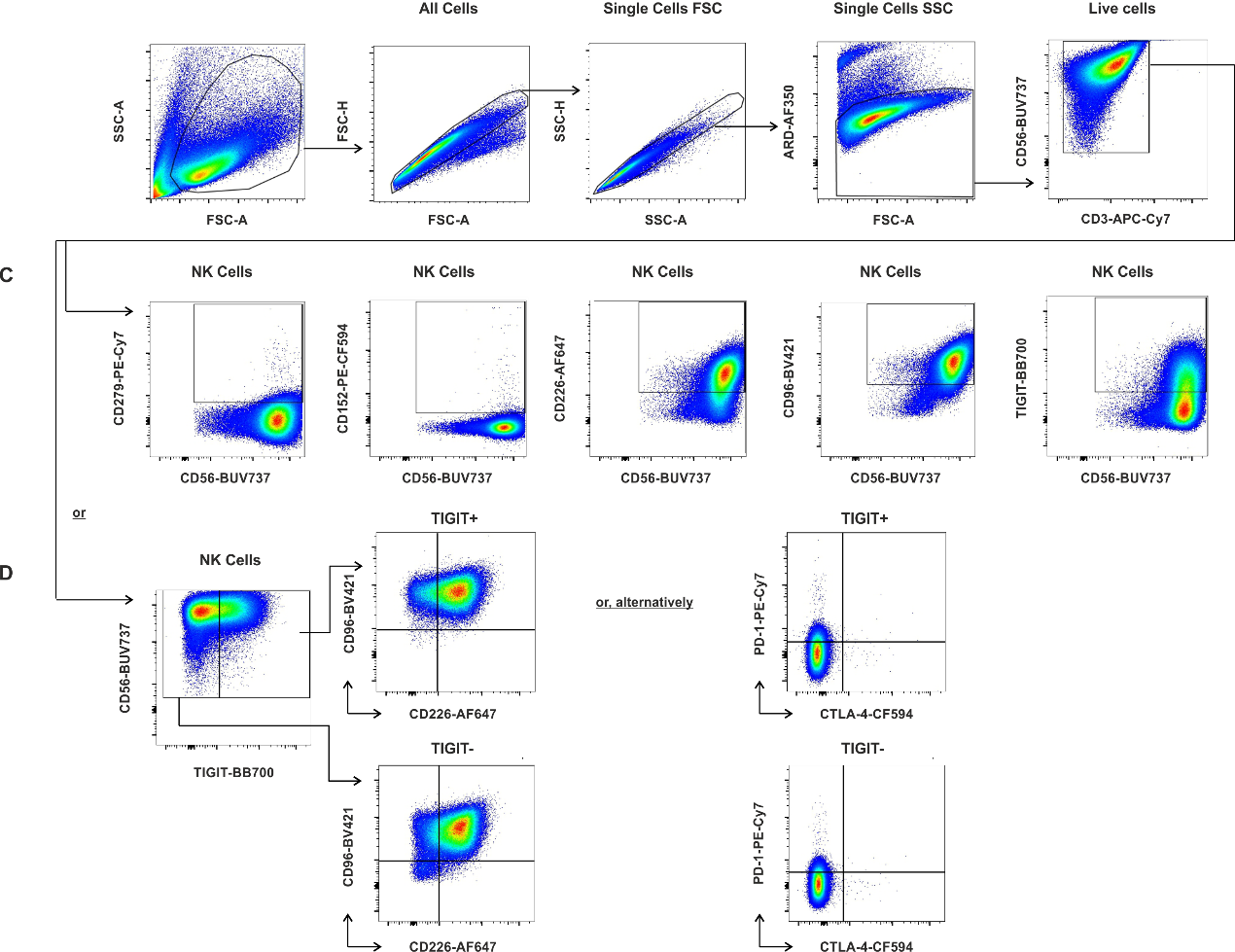


Suppl. Fig 2. Gating strategies. **A** and **C** Flow cytometric analysis allowing the identification of NK cell-specific receptor expression exemplified on **A** CIML-NK cells of donor SNK290S and **C** NK-92 cells. **B** and **D** Flow cytometric analysis allowing the identification of single-, double- or triple-positive NK cell subsets on **B** CIML-NK cells of donor SNK279B and **D** NK-92 cells.

**
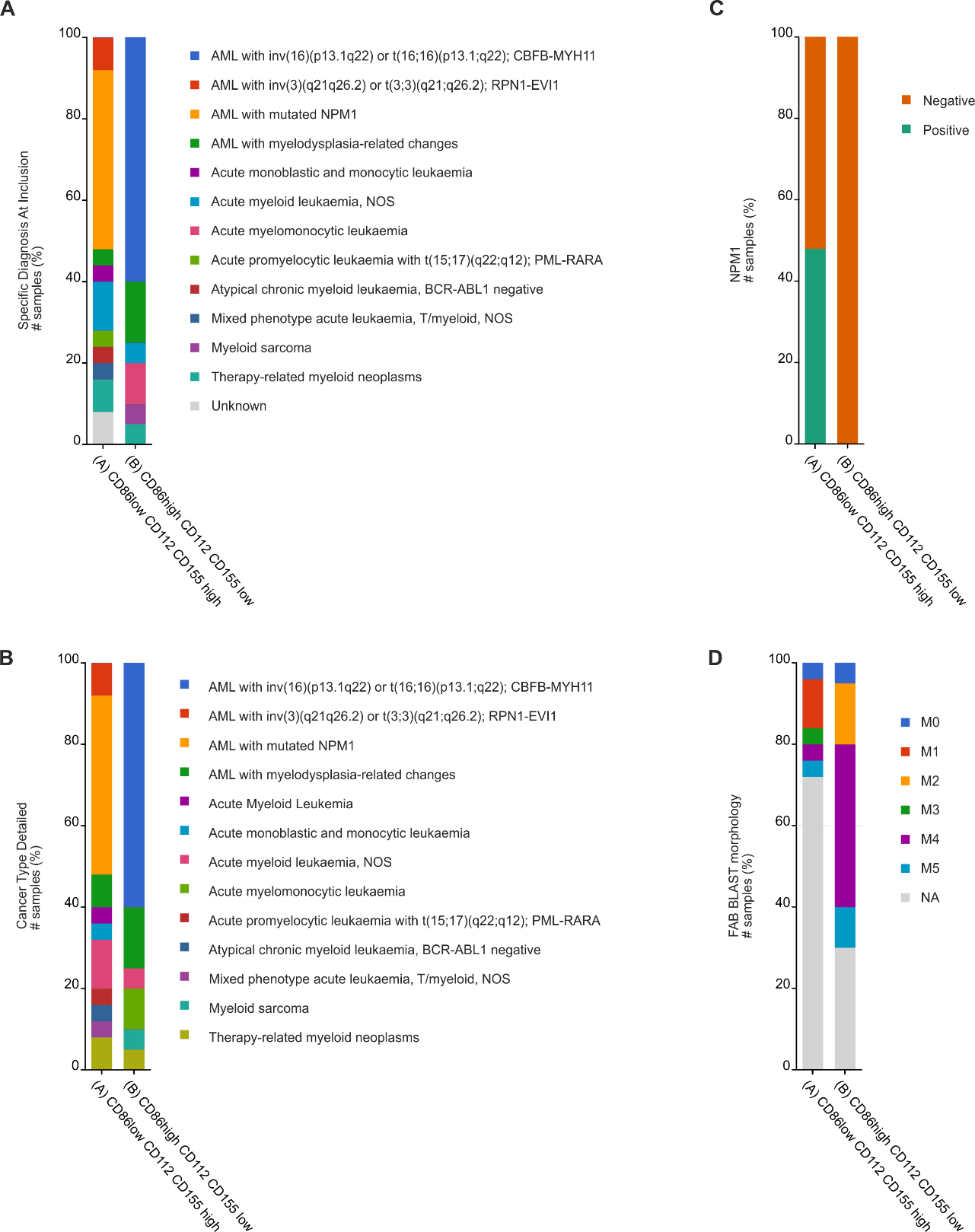
**

Suppl. Fig. 3. Distribution of cancer type and morphology in AML patients included in Fig. 3c. Shown are the two groups with a CD86^low^ CD112/155^high^ or a CD86^high^ CD112/CD155^low^ phenotype, respectively, for which significant differences in the cancer type or morphology (as indicated in the y-axis) exist. Data were obtained from the Beat AML 2.0 cohort dataset [1] and accessed via the cBioPortal [2]. The corresponding p-values are shown in Suppl. Tab. 3.

**Supplemental Literature**

1. Bottomly D, Long N, Schultz AR, Kurtz SE, Tognon CE, Johnson K et al. (2022) Integrative analysis of drug response and clinical outcome in acute myeloid leukemia. Cancer Cell. 40(8):850-864.e9.

2. Cerami E, Gao J, Dogrusoz U, Gross BE, Sumer SO, Aksoy BA, et al. (2012) The cBio Cancer Genomics Portal: An Open Platform for Exploring Multidimensional Cancer Genomics Data. Cancer Discov. 2(5):401–404.
